# Identification of Flavoanoids From Finger Citron and Evaluation on Their Antioxidative and Antiaging Activities

**DOI:** 10.3389/fnut.2020.584900

**Published:** 2020-10-22

**Authors:** Xuguang Luo, Jin Wang, Haiqiang Chen, Aimei Zhou, Mingyue Song, Qingping Zhong, Hanmin Chen, Yong Cao

**Affiliations:** ^1^Guangdong Provincial Key Laboratory of Nutraceuticals and Functional Foods, College of Food Science, South China Agricultural University, Guangzhou, China; ^2^Guangdong Zhancui Food Co., Ltd, Chaozhou, China

**Keywords:** finger citron, flavonoids, antioxidation, anti-aging, *Caenorhabditis elegans*

## Abstract

Finger citron (*Citrus medica* L. *var. sarcodactylis* Swingle) is a traditional Chinese herb and considered as a healthy food. Flavonoids are the major bioactive substances in Finger citron. In this study, the major flavonoids of finger citron (FFC) were purified with AB-8 macroporous resins, and then three of them were identified as diosmetin-6-8-di-*C*-glucoside, hesperidin and diosmetin-6-*C*-glucoside, and other two were preliminarily inferred as limocitrol 3-alpha-l-arabinopyranosyl-(1->3)-galactoside and scutellarein 4′-methyl ether 7-glucoside by high-performance liquid chromatography and ultraperformance liquid chromatography to quadrupole time-of-flight mass spectrometry. Further, their antioxidation and antiaging activities were determined *in vitro* and *in vivo*. *In vitro*, chemical assays revealed that the purified FFC had strong antioxidative activity as demonstrated by its strong DPPH (2,2-diphenyl-1-picrylhydrazyl) and ABTS [2,2-azinobis (3-ethyl-benzothiazoline-6-sulphonic acid) diammonium salt] radical scavenging activities and ORAC (oxygen radical absorbance capacity). *In vivo*, the purified FFC significantly increased the mean and maximum lifespan of *Caenorhabditis elegans* by 31.26 and 26.59%, respectively, and showed no side effects on their physiological functions. Under normal and oxidative stress conditions, purified FFC reduced the accumulation of reactive oxygen species (ROS) and malondialdehyde, while increased superoxide dismutase (SOD) and catalase (CAT) enzyme activities in *C. elegans*. Together, we successfully identified three major substances in purified FFC of finger citron and determined the excellent antiaging activity of FFC, which is attributed to its strong antioxidative activity and effect on homeostasis of ROS.

## Introduction

Flavonoids are important plant secondary metabolites presented broadly in nature (1), which provide protection against foreign agents such as UV radiation, parasite, and viruses (2). Currently, more than 9,000 flavonoids have been discovered (3), and *Citrus* fruits are found to be a rich source of flavonoids (4). The biological activity of flavonoids has been widely investigated, including antioxidative (5, 6), antiaging (6), anti-inflammatory (7), anticancer (8), antidiabetic, and antibacterial (9, 10).

Finger citron (*Citrus medica* L. *var. sarcodactylis* Swingle), with a popular name “Fo-Shou” in Chinese, belongs to the variants of *Citrus medica* L. and is considered as both food and medicine in China. As a food, finger citron is widely produced into preserved fruit (11). As a traditional Chinese medicine adjuvant, finger citron is often used for the treatment of stomachache, headache, infectious hepatitis, arthritis, etc. (12). Finger citron is now widely distributed in the southwest of China, India, Vietnam, and Malaysia (13) and has been reported to contain various bioactive components including essential oil (12), flavonoids (13), polysaccharide (14), coumarin, and phenolic acids (15), etc. The essential oil of finger citron has attracted many scientific interests due to its diverse bioactivities, such as anti-inflammatory, antioxidation, and antibacterial (12, 14, 16), but finger citron flavonoids, also as the primary bioactive ingredients in finger citron, received relatively few studies. Little information about the major components and biological activities of flavonoids from finger citron is available, which limits the exploration of their potential application value.

Aging has many adverse effects on human health. It increases the risk of cancer, neurodegenerative disorders, and cardiovascular and metabolic diseases (17–21). Reactive oxygen species (ROS), produced by all aerobic cells (22), play an important role in aging. According to the free radical theory of aging, which is later called oxidative stress theory of aging, age-related functional losses are caused as the result of the accumulation of oxidative damage to biological macromolecules by ROS and NO (22). Flavonoids have been reported to show excellent antioxidative and antiaging activities (23). The flavonoids extracted from finger citron may also have these functional activities. DPPH (2,2-diphenyl-1-picrylhydrazyl), ABTS [2,2-azinobis (3-ethyl-benzothiazoline-6-sulphonic acid) diammonium salt] radical scavenging capacity and ORAC (oxygen radical absorbance capacity) assays are the most commonly used assays for determining antioxidation performance *in vitro* (24), whereas *Caenorhabditis elegans* is a well-established *in vivo* model organism that has been successfully used to study organismal aging, antioxidation, and identification of new pharmacology (25, 26) because of its advantages of facile lifespan, ease of cultivation, complete genome sequence, and so on (27). Moreover, it is demonstrated that *C. elegans* possesses 60 to 80% of human gene homologs (28).

In the present study, flavonoids of finger citron (FFC) were extracted by continuous phase-transition extraction with a novel type of extraction device developed by us, which has great advantages in extraction efficiency and yield of active compositions (29, 30). The resulted FFC were then purified and identified, and its antioxidative and antiaging were further investigated *in vitro* and *in vivo*.

## Materials and Methods

### Materials

Dry finger citron slices were supported by Guangdong Zhancui Food Co., Ltd. (Chaozhou, Guangdong, China). The samples were further dried with hot air at 40°C for 24 h to guarantee the moisture content was lower than 15%. Then the dried samples were smashed into powder with particle size of 30 mesh, which was packed immediately in vacuum polyethylene bags and frozen at −20°C for further use.

### Chemicals

AB-8 macroporous resin was provided by Bengbu Tianxing Ion-Resin Co., Ltd. (BengBu, Anhui, China). DPPH and fluorescein sodium salt were purchased from Sigma Chemical Co., Ltd. (St. Louis, United States). Trolox, ABTS, and 2,2′-azobis(2-methylpropionamidine) dihydrochloride (AAPH) were purchased from Aladdin Bio-Chem Technology Co., Ltd. (Shanghai, China). *C. elegans* of wild-type N2 (var. Bristol) was obtained from the Caenorhabditis Genetics Center (University of Minnesota, Minneapolis, MN, United States). The uracil mutant *Escherichia coli* OP50 (*E. coli* OP50) was provided by the College of Resource and Environment, South China Agricultural University. 2′,7′-dichlorodihydrofluorescein diacetate (H_2_DCF-DA) and paraquat (PQ) were purchased from Sigma–Aldrich Co., Ltd (St. Louis, MO, USA). SOD (superoxide dismutase), CAT (catalase), and MDA (malondialdehyde) assay kits were provided by Nanjing Jiancheng Bioengineering Institute (Nanjing, Jiangsu, China). All other chemicals were of analytical-reagent grade.

### Isolation and Purification of FFC

Based on our previous study, 400 g of finger citron dry powder was used to extract crude FFC with 85% ethanol by continuous phase-transition extraction at 0.2 MPa and 90°C for 120 min. AB-8 macroporous resin was then chosen from four types of macroporous resins (AB-8, D-301, HPD-300, HPD-400) to purify the resulted crude FFC by column chromatography conducted on a glass column (2.4 × 30 cm) according to the parameters determined in advance. Briefly, 1,080 mL FFC solution (2 mg total flavonoids per mL) was loaded onto the column with AB-8 (bed volumes 135 mL) at 4 mL/min and kept at room temperature for 270 min to reach adsorption equilibrium. The saturated resin was then eluted with 1,400 mL of 60% ethanol at 0.4 mL/s. The collected fraction was concentrated by a rotary evaporator (R204B3, Shanghai Shensheng Technology Co., Ltd., Shanghai, China) at 50°C, followed by freeze-drying with a lyophilizer (FD-1PF, Beijing DETIANYOU Instrument Co., Ltd., Beijing, China) and stored at −20°C for further use. The purified FFC contain 50.5% flavonoids.

The flavonoid content in the purified FFC was determined by UV-vis spectrophotometer (UV-3010, HITACHI, Japan) at the wavelength of 420 nm according to the method of NY/Y 2010–2011 (an officially recognized Chinese criterion for the determination of total flavonoids in citrus fruits and derived products). A standard curve was established using hesperidin with the concentration range from 0.00 to 0.10 mg/mL. Briefly, 1.00 mL purified FFC liquid was diluted to 5.00 mL with a blank solution reagent (NaOH-citric acid buffer solution, pH 6.0) and then accurately mixed with 5.00 mL of “9+1” diethylene glycol solution (900 mL diethylene glycol + 100 mL distilled water) and 0.10 mL of 160 g/L NaOH solution in 10 mL colorimetric tube. At the same time, the same amount of test solution without NaOH solution was used as absorbance of a reagent blank. The mixture was put in a water bath at 40°C for 10 min and cooled in a cold-water bath for 5 min. The content of flavonoids in the purified FFC was calculated based on the following equation:

(1) $$\begin{array}{l} \text{Purified FFC flavonoids content}=\frac{c\times \text{v}\times f}{m}\times 100\% \end{array}$$

where *c* (mg/mL) was the hesperidin concentration calculated according to the standard curve; *v* (mL) was the sample liquid volume; *f* was dilution factor of the sample solution; and *m* (g) was the weight of the purified FFC after freeze-drying.

### Chemical Analysis of Major Peak Components of FFC

High-performance liquid chromatography (HPLC) and ultraperformance liquid chromatography to quadrupole time-of-flight mass spectrometry (UPLC-Q-TOF-MS) were used for qualitative identification of the major peak components in purified FFC. For HPLC analysis, an LC-10AT VP plus system (Shimadzu, Kyoto, Japan) concentration with an Eclipse Plus C_18_column (250 × 4.6 mm, 5 μm, Agilent) was used to analyze purified FFC. The mobile phase constituted 0.1% formic acid solution in water (A) and methanol (B), the gradient of the mobile phase was as follows: 0–40 min, 25–80% B, 40–45 min, 80–95% B, 45–70 min, 95–25% B. The flow rate was 1 mL/min. The detection wavelength was 280 nm, and the injection volume was set as 20 μL.

UPLC separation was carried out using an Eclips plus C_18_ column (100 × 2.1 mm, 1.8 μm, Agilent) with gradient solvents A and B in mobile phase, where A was 0.4% formic acid (vol/vol) in distilled water and B was acetonitrile with a gradient of 25% to 60% in 20 min at a flow rate of 1.0 mL/min. The injection volume was 10 μL, and the UV detection wavelength was 280 nm. An Agilent 6540UHD Q-TOF tandem mass spectrometer was used for MS and MS/MS detection. The operation conditions were as follows: drying gas (N_2_) flow rate, 8 L/min; drying gas temperature, 300°C; nebulizer, 50 psig; sheath gas flow rate, 12 L/min; sheath gas temperature, 350°C; capillary voltages, 4,000 V; fragmentor, 130 V; skimmer, 65 V; OCT RF Vpp, 750 V. The data were acquired in negative ion mode; mass spectra were recorded across the range of m/z 105–1,100.

### Antioxidant Activities of FFC Against DPPH Radical

DPPH radical-scavenging activity of the purified FFC was determined using the mothod of Najafian et al. (31) with some modifications. Blank ethanol solvents or flavonoid sample solutions (100 μL) were mixed with a 100 μL DPPH (2 × 10^−4^ mol/L) ethanol solution in a 96-well plate. The mixtures were incubated for 30 min at room temperature in the dark. The absorbance was read at 517 nm. Ascorbic acid was used as a positive control. The scavenging capability against DPPH radical was calculated as follows:

(2) $$\begin{array}{l} \text{DPPH radical}-\text{scavenging activity }\left(\text{\%}\right)\\ \;=\left[1-\left({\text{A}}_{t}-{\text{A}}_{r}\right)/{\text{A}}_{0}\right]\times 100 \end{array}$$

Where A_t_ represents the absorbance of the sample at 517 nm, A_r_ was the absorbance of sample solution and A_0_ was the absorbance of DPPH solution.

### Antioxidant Activities of FFC Against ABTS Radical

ABTS radical-scavenging activity of the purified FFC was determined according to the method of Re et al. (32) with slight modifications. ABTS radical cation (ABTS^+^) was produced by mixing 5 mL ABTS stock solution (7 mmol/L) with 88 μL potassium persulfate (140 mmol/L) and allowing the mixture to stand at room temperature for 12 h in the dark. Distilled water was then mixed with ABTS^+^ to each measurement until an absorbance of 0.70 ± 0.02 was shown at 734 nm. A 100 μL of the ABTS^+^ solution was allowed to react with 100 μL of samples at different concentrations or 75% ethanol (as a control) or ascorbic acid (as a positive control) for 10 min. Absorbance was measured at 734 nm immediately by Microplate reader (Enspire2300, PE, US). The scavenging capability against ABTS radical was calculated as follows:

(3) $$\begin{array}{l} \text{ABTS radical}-\text{scavenging activity }\left(\text{\%}\right)\\ \;=\left[1-\left({\text{A}}_{\text{t}}-{\text{A}}_{\text{r}}\right)/{\text{A}}_{0}\right]\times 100 \end{array}$$

Where A_*t*_ represents the absorbance of the sample at 734 nm, A_*r*_ represents the absorbance of the mixtures of sample and ethanol, A_0_ represents the absorbance of the control.

### Measurement of ORAC

The ORAC assay was carried out following the method of Thaipong et al. (24) with a slight modification to determine the peroxy radical scavenging activity of the purified FFC. Briefly, 100 μL of fluorescein (8.4 × 10^−8^ mol/L) in 75 mmol/L phosphate buffer (pH 7.4) was added to the wells of a 96-well plate Fluorescein was measured to determine the background excitation at 490 nm and emission at 514 nm. After that, 50 μL of the sample, Trolox standard or blank (distilled water) was added to the wells of the 96-well plate, followed by oscillation for 3 min and incubation at 37°C for 10 min. Subsequently, 50 μL of AAPH (153 mmol/L, freshly prepared in phosphate buffer) was added to each well, and the fluorescence value was measured every 90 s in 3.5 h. The final ORAC value was calculated from the net area (between the Trolox standard curve and the blank value) and the regression equation for different concentrations of Trolox (0.02, 0.04, 0.06, 0.08, and 0.1 mmol/L). The ORAC value was expressed as the average micromolar ± SD of the Trolox equivalent (TE) per micron of compound.

### Cultivation and Synchronization

*Caenorhabditis elegans* were cultured and assayed on NGM plates at 20°C and *E. coli* OP50 bacteria were inoculated. Synchronized hermaphrodite population were obtained by the sodium hypochlorite treatment, which killed the adult worms and recovered the hatched L1 larvae on NGM/OP50 plate (33).

### Preparation of Treatment Plates

A 5 μL of purified FFC (200 μg/mL) and control (distilled water) were filtered to remove bacteria, mixed with 95 μL *E. coli* OP50, and then inoculated onto NGM (65 mm × 10 mm) plates to feed worms.

### Lifespan Assay

The lifespan studies were performed as previously described (34). Synchronized L4 larvae were further transferred to fresh NGM plates with 5 μL of purified FFC (200 μg/mL) and control (distilled water) (3 plates per group, and at least 30 L4 larvae of N2 wild type per plate). During the reproductive period, the worms were transferred to the NGM plates every day. In other periods, the worms were transferred to new plates every 2 days to ensure the concentration of the treated compounds. Surviving and dead worms (the criterion for worm death was that there was no respond to a touch-provoked) were counted every alternate day from the first day of L4 larvae until all worms had died. The worms should be excluded from the statistics, which escaped from NGM plates or suffered from hatching of embryos within the adult hermaphrodite before eggs were laid (a so-called “internal hatch”).

### Reproduction Assay

The reproduction assay was determined using the method of Chen et al. (35) Briefly, the L4 larvae of *C*. *elegans* (10 individuals, 2 worms per plate) were shifted to fresh plates every day during reproduction day until worms were basically no longer spawning. Eggs on all plates were allowed to hatch at 20°C for 24 h and the number of progenies of each worm was counted at L4 stage.

### Movement Assay

This assay was performed according to the previously reported method with a slight modification (36). The worms were cultured from eggs similar to the method described in the lifespan assay. On days 5, 10, and 15 of life, 10 individuals were randomly selected and their body locomotion phenotype were measured by gently prodding them with a platinum wire. Worms that move spontaneously and smoothly in a sinusoidal and symmetrical pattern were categorized as class A. Worms that respond to touch but move slowly and uncoordinatedly were classified as class B. And worms which move their noses or tails only when prodded were classified as class C. Meanwhile, the sinusoidal locomotion of the worms was also analyzed by recording the number of worms that move in a sinusoidal manner within 1 min on the 5th, 10th, and 15th days (7 worms were selected from each plate for recording), while the head swing frequency was measured on days 2 and 6 by transferring the L4 stage worms into each set of medium and the standard of the head swing frequency was the number of times from one side to the other side within 30 s.

### Thermotolerance and Oxidative Stress Assays

Thermotolerance and oxidative stress assays were carried out based on the method described by Hansen et al. (37) with a slight modification. Thermotolerance assay was performed with hermaphrodites at 20°C as the aging assay. After 96-h treatment with purified FFC, the adult worms (30 worms per plate) were changed from 20 to 35°C. Survivals were scored every 4 h to draw the life curve after the temperature changed and the worms that died due to dryness on both sides of the plate were excluded. For oxidative stress assay, the worms were transferred into the newly prepared NGM/*E. coli* OP50 plates containing paraquat (25 mg/mL, M/V) after treatment with purified FFC or control for 96 h. The worm survival rate was recorded every 24 h until all of the worms died (the worms did not respond when touched with a platinum wire). In addition, ROS, SOD, CAT, and MDA were determined according to the methods described in Measurement of ROS and Measurement of SOD, CAT, and MDA after oxidative stress treatment.

### Measurement of ROS

ROS detection was performed as the reported literature (35). Synchronized L4 larvae were transferred to fresh NGM plates with 5 μL of purified FFC (200 μg/mL) or control (distilled water). After treatment with purified FFC or control for 96 h, bacteria were removed by transferring the worms to the new NGM plate 3 times. Eighty worms were then transferred into a 96-well plate with 50 μL of M9 buffer. Meanwhile, 50 μL of 100 μM H_2_DCF-DA solution in M9 buffer was added to the 96-well plate including a worm-free control well containing H_2_DCF-DA. The fluorescence intensity was measured by an EnSpire® Multimode Plate Reader (PerkinElmer, USA) every 20 min for 20 h at a reaction temperature of 25°C, an emission wavelength of 528 nm and an excitation wavelength of 485 nm. The results were presented as relative fluorescence units (RFU).

### Measurement of SOD, CAT, and MDA

SOD, CAT and MDA detection were conducted according to the method described by Liu et al. (38) with a minor modification. After treatment with purified FFC or control for 96 h, 200 worms were rinsed three times with sterile water and centrifuged at low speeds. The supernatant was then discarded and the precipitate was adjusted with 0.5 mL sterile water, followed by ultrasonically decomposition with a JY92-2D Ultrasonic cell Disruption System (Scientz, China) for 2 min (repeated twice) at low temperature. Subsequently, 0.5 mL of 1% CHAPS solution (wt/vol) was added to the worm homogenate and centrifuged for 15 min (4°C, 12,000 rpm). The supernatant was gathered and stored at 4°C until use. SOD, CAT and MDA were, respectively, detected with SOD assay kit (WST-1 method), CAT assay kit (Ultraviolet), and MDA assay kit (Colorimetric method) (Nanjing Institute of Bioengineering Institute, China). Enzyme activities were expressed in units of milligrams of protein.

### Statistical Analysis

All results were presented as mean ± SD (*n* = 3). One-way analysis of variance (ANOVA) was performed using SPSS 16.0 software (SPSS Inc., Chicago, IL, United States), and different letters indicate that the values were significantly different (*p* < 0.05).

## Results and Discussion

### Chemical Analysis of the Major Peak Components of FFC

**Figure 1:**
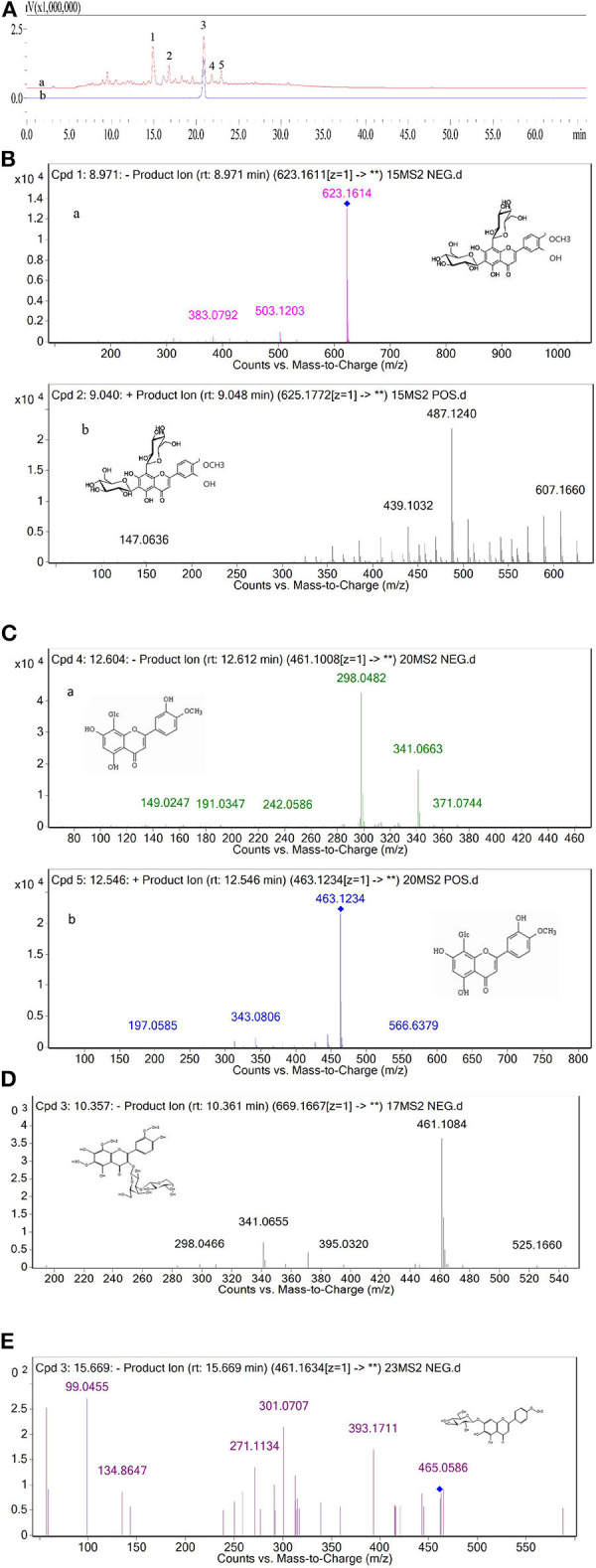
**(A)** HPLC chromatogram of the purified FFC detected at 280 nm (a) and hesperidin standard detected at 280 nm (b). **(B)** The MS/MS of peak 1 in negative ionization (a) and positive ionization (b). **(C)** The MS/MS of peak 4 in negative ionization (a) and positive ionization (b). **(D)** The MS/MS of peak 2 in negative ionization. **(E)** The MS/MS of peak 5 in negative ionization.

On the basis of our previous work, crude FFC prepared by continuous phase transition extraction were first purified by AB-8 resin. Five components (1–5) of the purified FFC were found to have high UV absorption at 280 nm as shown in the HPLC spectrogram (Figure 1A-a). According to the reported literature (39), the major flavonoids in *Rutaceae* family were hesperidin and naringin. Therefore, peak 3 in the HPLC spectrum of the purified FFC was confirmed as hesperidin by comparing to hesperidin standard substance. Other peaks (1, 2, 4, 5) obtained by HPLC were further analyzed by UPLC-Q-TOF-MS/MS. Based on the previous study on the MS spectra of these flavonoids, these peaks were identified by generating molecular formula and the fragmentation pattern in positive and negative ionization modes as depicted in Table 1 and Figures 1B–E. Peak 1 with a molecular anion at 623 and typical *C*-glycosyl fragments at m/z 503, 383 (M-H-120, M-H-240) was assigned as diosmetin 6,8-di-*C*-glucoside with a molecular formula C_28_H_32_O_16_ (40). Peak 4 with [M-H]^−^ ion at m/z 461 ([M+H]^+^ ion at m/z 463) and characteristic MS^n^ ions at 341(343) (M-120) and 298 was identified as diosmetin 8-*C*-glucoside (orientin 4′-methyl ether) with a molecular formula C_22_H_22_O_11_ (41). Peak 2 and 5 [(M-H)^−^ ions at m/z 669 and 461, respectively], were preliminarily judged as limocitrol 3-alpha-l-arabinopyranosyl-(1->3)- galactoside (MS^n^ ions at m/z 461, 395, 341) and scutellarein 4′-methyl ether 7-glucoside (MS^n^ ions at m/z 393, 301, 271) by molecular structure correlation, respectively.

**Table 1:** Identification of the major compounds in the purified FFC by UPLC-Q-TOF-MS/MS.

**Peak No**.	**Rt (min)**	**Formula**	**[M-H]^**−**^/[M+H]^**+**^ (m/z)**	**MS^**n**^ Inos (–/+) (m/z)**	**Tentative identification**	**Reference**
1	14.875	C_28_H_32_O_16_	623.1611/625.1767	533.1301,503.1203, 383.0792/607.1660, 487.1240,439.1032	Diosmetin-6-8-di-*C*-glucoside	(40)
2	16.775	C_29_H_34_O_18_	669.1667/	461.1084,395.0320, 341.0655/	Limocitrol 3-alpha-L-arabinopyranosyl-(1->3)-galactoside	
3	20.884	C_28_H_34_O_15_	–	–	Hesperidin	By hesperidin standard
4	21.816	C_22_H_22_O_11_	461.1088/463.1236	341.0663, 298.0482/343.0811	Diosmetin-6-*C*-glucoside	(41)
5	22.933	C_22_ H_22_O_11_	461.1634/	393.1711,301.0707, 271.1134/	Scutellarein 4′-methyl ether 7-glucoside	

### *In vitro* Antioxidant Activity of FFC

**Figure 2:**
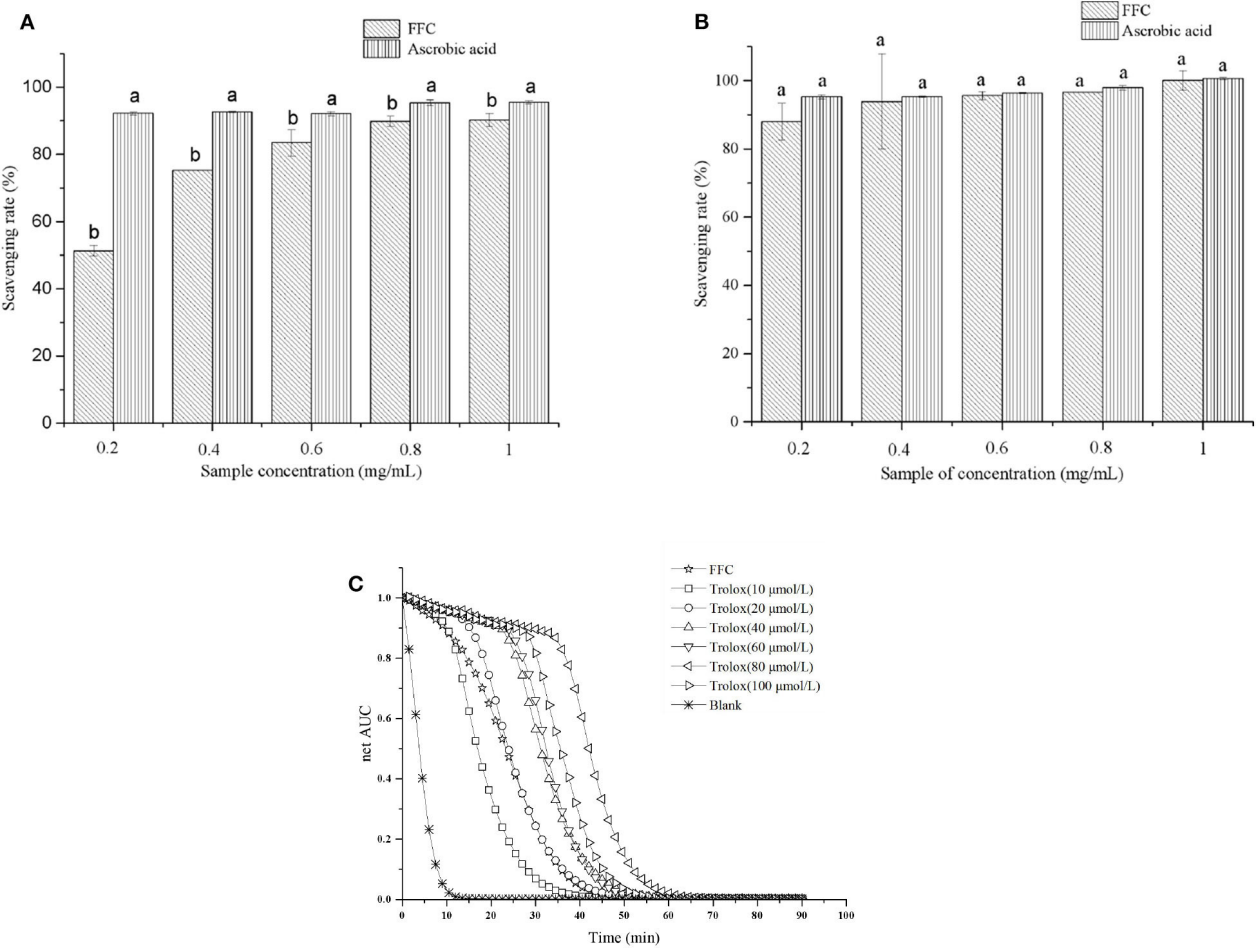
*In vitro* antioxidant tests for the purified FFC. DPPH scavenging activity **(A)**, ABTS scavenging activity **(B)**, and fluorescence decay curves **(C)**. Different letters indicate that the values were significantly different (*p* < 0.05).

DPPH, ABTS scavenging capacity and ORAC assay are the most commonly used methods for determining antioxidation performance *in vitro* (24). The scavenging capacity of DPPH and ABTS radicals of the purified FFC were investigated at concentrations of 0.2 to 1 mg/mL and compared with ascorbic acid, which served as a control. As shown in Figures 2A,B, the purified FFC exhibited strong scavenging capacity for both DPPH and ABTS radicals. Interestingly, FFC showed more potent effect to scavenge ABTS radical than that to DPPH radical at the same concentration. In addition, the DPPH radical scavenging ability of the purified FFC was in a dose-dependent manner (0.2–0.8 mg/mL, *p* < 0.05), and its scavenging ability was close to that of ascorbic acid at the concentration of 0.8 mg/mL, which was 89.86% and up to 93.68% of that of ascorbic acid (*p* < 0.05). At 1.0 mg/mL, the DPPH radical scavenging ability of the purified FFC was 90.24%, which exhibited no significant difference with that of 0.8 mg/mL (*p* > 0.05) and amounted to 94.74% of that of ascorbic acid (*p* < 0.05). For ABTS radical scavenging ability, the purified FFC had a strong scavenging capacity of 87.94% at low concentration (0.2 mg/mL) and the increase of the concentration had no evident effect on its capacity (*p* > 0.05). Furthermore, the ABTS^+^ scavenging ability of the purified FFC was not significantly different from that of ascorbic acid at concentrations of 0.2 to 1 mg/mL (*p* > 0.05). Figure 2C depicted the ORAC value of the purified FFC. The results demonstrated that the purified FFC exhibited strong antioxidative activity with an ORAC value of 928.64 μmol TE/g, which was equal to that of 20 μmol/L Trolox.

DPPH and ABTS assays are based on an electron transfer and involve reduction of a colored oxidant (24, 42), while ORAC assay involves the transfer of a hydrogen atom, in which substrate and antioxidants compete for thermally created peroxyl radicals (43). Determination of these antioxidant capacities *in vitro* are helpful to investigate the potential health benefits of the purified FFC on oxidative stress mediated diseases (e.g., aging, inflammation) (42). Chanput et al. (44) tested the antioxidative activity of three sub groups of flavonoids at a range of concentrations by *in vitro* chemical-based assays including DPPH, ABTS, and ORAC methods and found the antioxidative activities of these flavonoids were dose-dependent and in opposite relationship with the decrease of inflammatory genes. Our results demonstrated that the purified FFC had strong antioxidative function *in vitro*, suggesting it may potentially have excellent antiaging activity.

### Effects of FFC on the Lifespan of *C. elegans*

**Figure 3:**
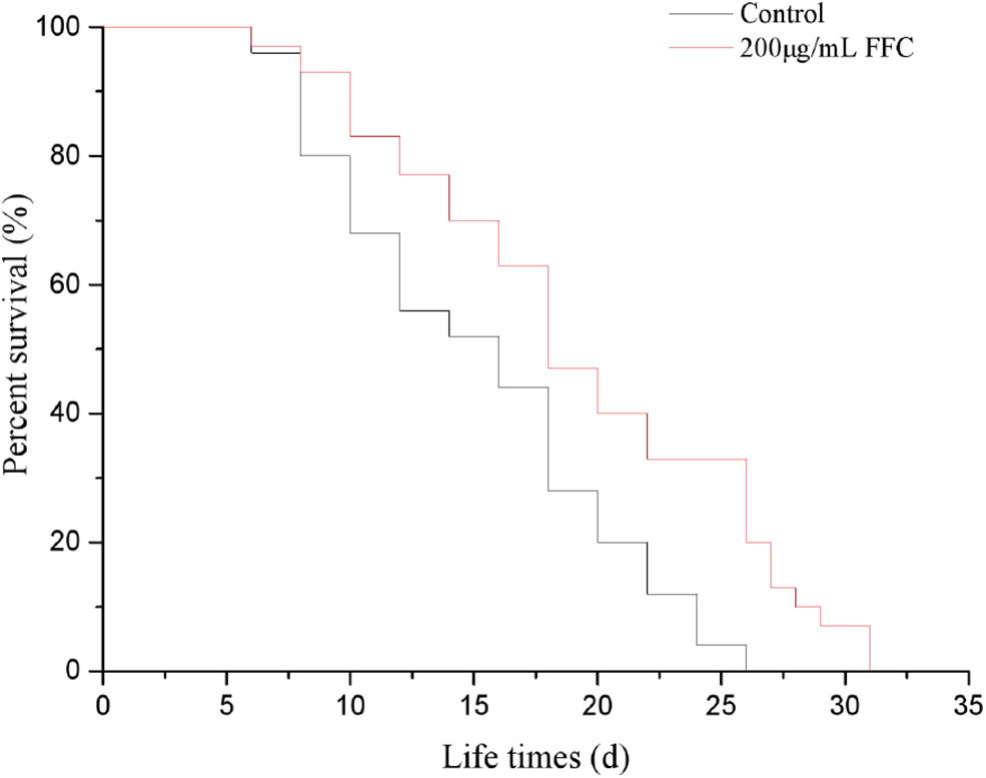
Effects of the purified FFC on the fraction survival of *C. elegans*. Survival curves of wild-type (N_2_) worms raised at 20°C on the plates containing H_2_O (control) or the purified FFC (200 μg/mL). The survival curves treated with 200 μg/mL of the purified FFC were significantly different by the log-rank test (*p* < 0.001).

It is well-confirmed that irreversible oxidative damage accumulates during aging (45). To understand the antioxidative and antiaging effect of FFC, N2 wide-type worms were treated the purified FFC started from L4 larvae till death. Based on our previous study, 200 μg/mL purified FFC was the optimal dose on lifespan elongation of *C. elegans*, so 200 μg/mL was selected for our later experiments. As depicted in Figure 3 and Table 2, purified FFC not only manifested a notable survival curve (*p* < 0.001, by the log-rank test), but also evidently increased the mean lifespan and maximum lifespan of the worms (*p* < 0.05), which were 31.26 and 26.59% greater than those of the control, respectively. The total lifespan of *C. elegans* was about three to 4 weeks, and the extension of 1 or 2 days in the lifespan is significantly different (26), so these findings indicated that supplementation with the purified FFC could significantly prolong the lifespan of *C. elegans*. As compared with the blank group, Cai et al. (46) also confirmed that the high dose *Epimedium* flavonoids group could significantly prolong the mean lifespan and maximum lifespan.

**Table 2:** Effects of the purified FFC on the lifespan of *C. elegans*.

**Concentration (μg/mL)**	**Mean of lifespan (mean ± SD) (days)**	**Maximum lifespan (mean ± SD) (days)**	**Mean fold increase (%)**
Control	15.66 ± 1.35^b^	16.96 ± 0.85^b^	0^b^
200 μg/mL FFC	20.56 ± 0.97^a^	21.47 ± 0.94^a^	31.26 ± 5.04%^a^

*Different letters in a column indicate that the values were significantly different (p < 0.05)*.

### Effects of FFC on Physiological Functions of *C. elegans*

**Figure 4:**
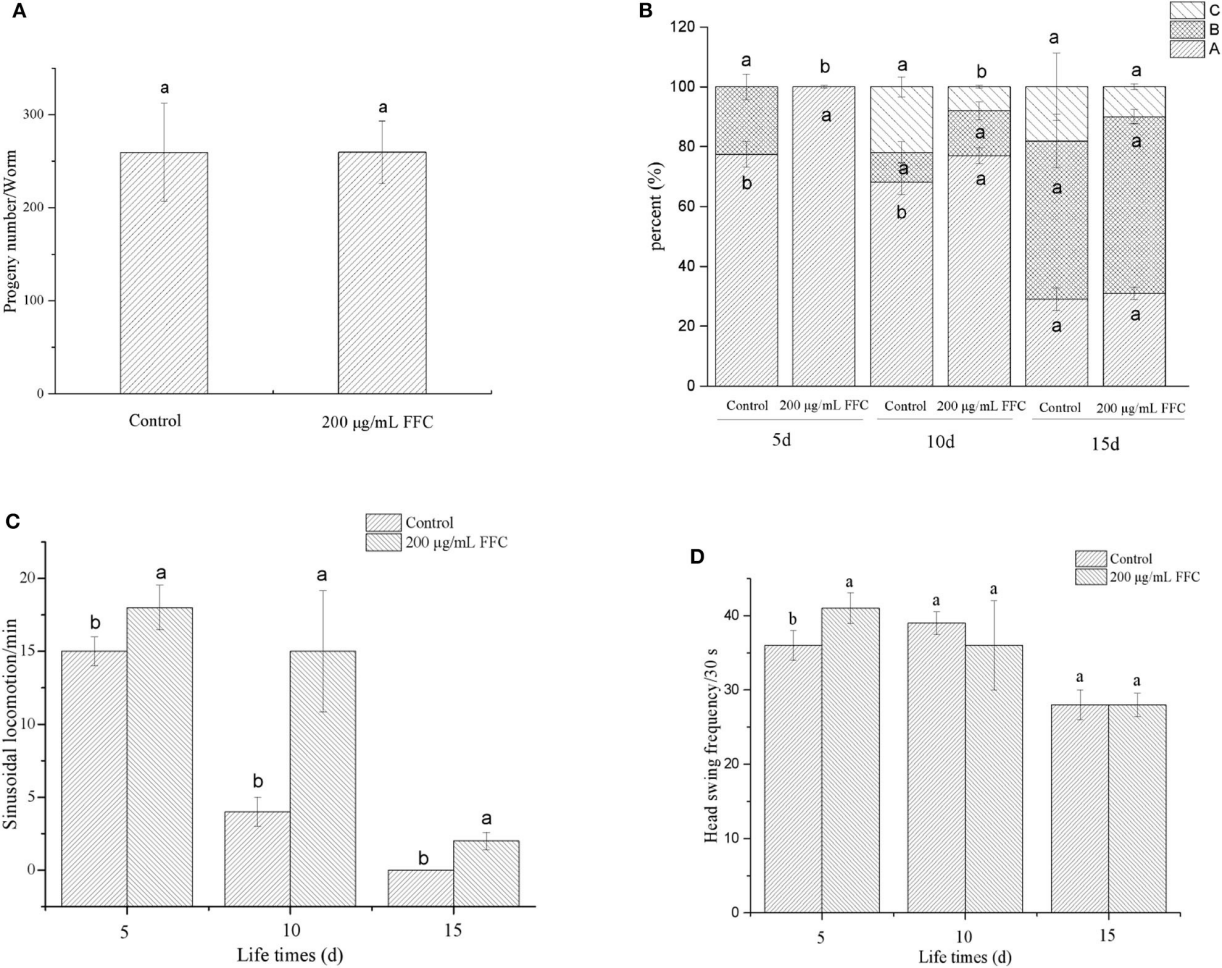
Effects of the purified FFC on the physiological functions of *C. elegans*. **(A)** The number of progeny of each worm was counted until the parental worms were dead or stopped producing progeny. The three levels of locomotivity **(B)**, the sinusoidal locomotion **(C)**, and the frequency of the head swing **(D)** were performed under a dissecting microscope for 30 s on days 5, 10, and 15 of the worms. Different letters in these groups denote that the values were significantly different (*p* < 0.05).

The aging of *C. elegans* is accompanied by a decline in physiological functions, such as reproduction, motility, and the response to external mechanical stimuli (47). Some mechanisms that extend lifespan have a side impact on their progeny production capacity (48). In our study, we recorded the effect of FFC treatment on the progeny production per day to investigate whether FFC suppressed or delayed nematode reproduction. Results showed that the purified FFC at 200 μg/mL neither slowed reproduction nor reduced total progeny production as illustrated in Figure 4A (*p* > 0.05). Interestingly, these findings indicated that FFC elongated the lifespan of *C. elegans* not by restraining or delaying progeny production. Similarly, Yang et al. (23) also confirmed that flavonoids from *Toona sinensis* leaf could improve the mean and maximum lifespan of *C. elegans*, but showed little effect on its reproductive capacity.

The aging of *C. elegans* is correlated with the level of muscle deterioration, which leads to the decline of move ability and response to external mechanical stimuli (47). To determine whether FFC can enhance lifespan of *C. elegans* by extending movement ability, different movement characteristics including the locomotivity, sinusoidal locomotion and head swing frequency were tested to evaluate the movement behavior at early, middle, and mid-late life stages (i.e., on the 5th, 10th, and 15th day, respectively). After treatment with 200 μg/mL of purified FFC, the movement of worms was scored using three classes of locomotion phenotypes (A, B, and C classification) as shown in Figure 4B. The control group appeared class B movement in early life stage (day 5), while the treatment of FFC could reduce the class B movement to keep the normal sinusoidal (A) movement. With the aging of *C. elegans*, class C began to appear in middle stage (day 10), and FFC could inhibit C-type movement, thereby improving its movement ability (*p* < 0.05). But there was no significant difference between the control and FFC group on locomotion ability in mid-late stage (*p* > 0.05). Figure 4C demonstrated that FFC treatment could also increase the sinusoidal locomotion of the worms at three life stages (*p* < 0.05), while Figure 4D showed that FFC could increase the head swing frequency of the worms significantly only in early stage when compared with the non-treated control worms (*p* < 0.05). These findings indicated that FFC could increase the movement ability of *C. elegans* to some extent, primarily in the early and middle stages.

To summarize, FFC could prolong the lifespan of *C. elegans* without causing obvious defect in its physiological functions, including reproduction, exercise capacity, and respond to external mechanical stimuli. Therefore, we continued to gain insight into the related mechanisms.

### Effects of FFC on the Stress Resistance of *C. elegans*

**Figure 5:**
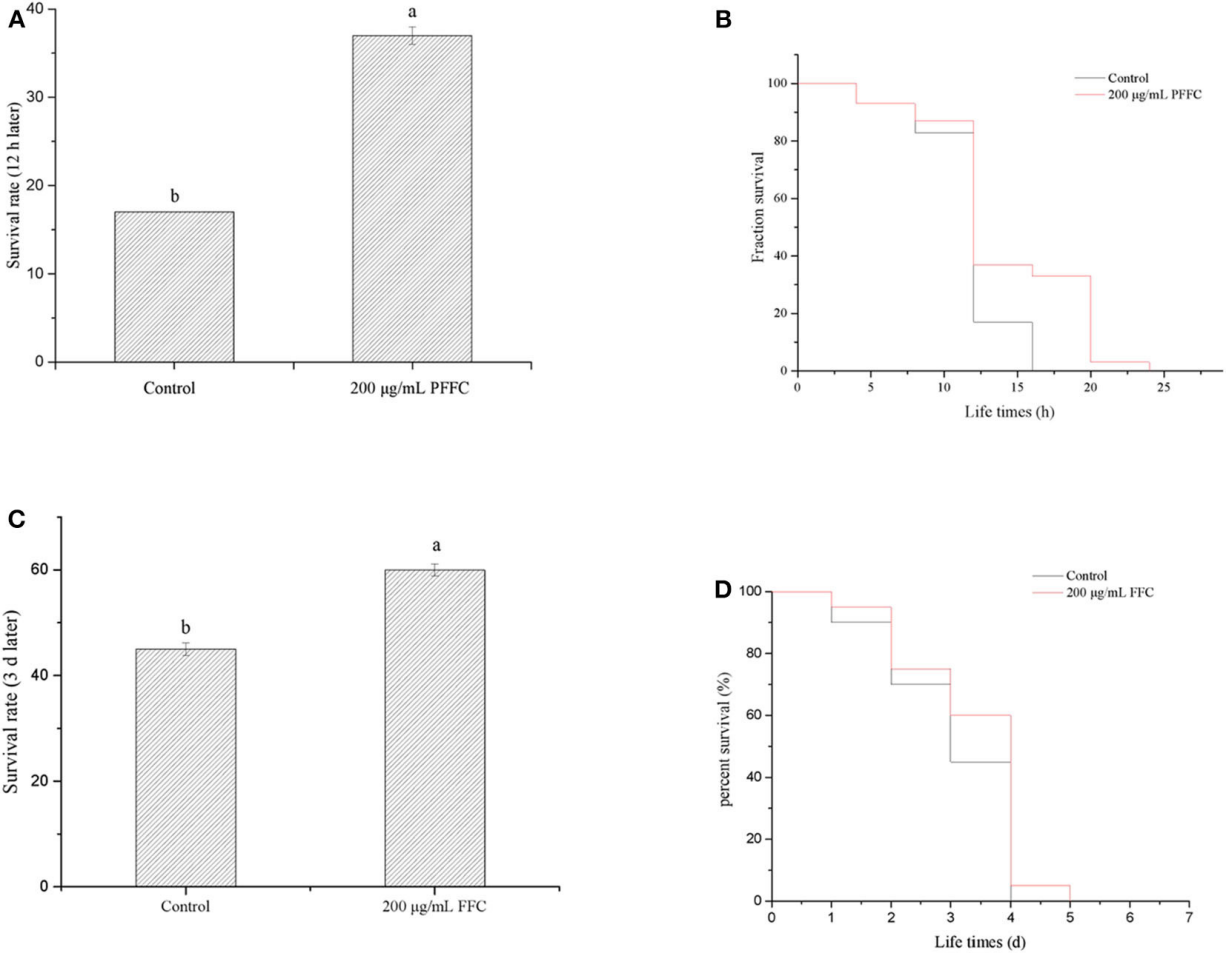
Effects of the purified FFC on thermal and oxidative resistance of *C. elegans*. **(A)** Survival rates of worms after 12 h when they were shifted from 20 to 35°C on day 4. **(B)** Survival curve of worms after they were shifted from 20 to 35°C on day 4. The survival curves of worms treated with 200 μg/mL purified FFC were significantly different by the log-rank test (*p* < 0.01). **(C)** Survival rates of worms after they were transferred into the freshly prepared NGM/OP50 plates containing paraquat (25 mg/mL) on day 3. **(D)** Survival curve of worms after they were transferred into the freshly prepared NGM/OP50 plates containing paraquat (25 mg/mL). Different letters indicate that the values were significantly different (*p* < 0.05).

According to the previous studies, longevity extension is usually associated with increased survival under stress conditions (49). The worms treated with or without FFC were shifted from 20 to 35°C on the fourth day to determine whether FFC has the effect of extending the lifespan of the worms under heat shock. As shown in Figure 5A, the survival rates of the worms treated with the purified FFC increased significantly from 17% (the control) to 37% after 8 h at 35°C (*p* < 0.05). In addition, FFC-treated worms showed a significantly different survival curve compared to the control group (*p* < 0.01 by the log-rank test) (Figure 5B). These findings declared that FFC supplements could act protectively under the thermal stress and enhance resistance to heat shock in *C. elegans*. Cai et al. (46) also found that *Epimedium* flavonoids could significantly prolong the mean lifespan of *C. elegans* under heat shock at 35°C.

Moreover, to determine whether purified FFC has the ability to extend worm lifespan under oxidative stress, the worms were transferred into the freshly prepared NGM/OP50 plates containing paraquat (25 mg/mL) after 96-h treatment with purified FFC or control. Although the survival curve of the worms treated by FFC was not significantly different from that of the control group (*p* > 0.05 by the log-rank test) (Figure 5D), their survival rates exhibited a significantly increase after 3 d compared to the control group, which increased by 33.33% as illustrated in Figure 5C, suggesting FFC had protective effects against paraquat-treated worms. These results suggested that FFC supplements had protective effects on *C. elegans* against paraquat-induced oxidative stress.

### Effects of FFC on Accumulation of ROS, MDA, and Enzyme Activity

**Figure 6:**
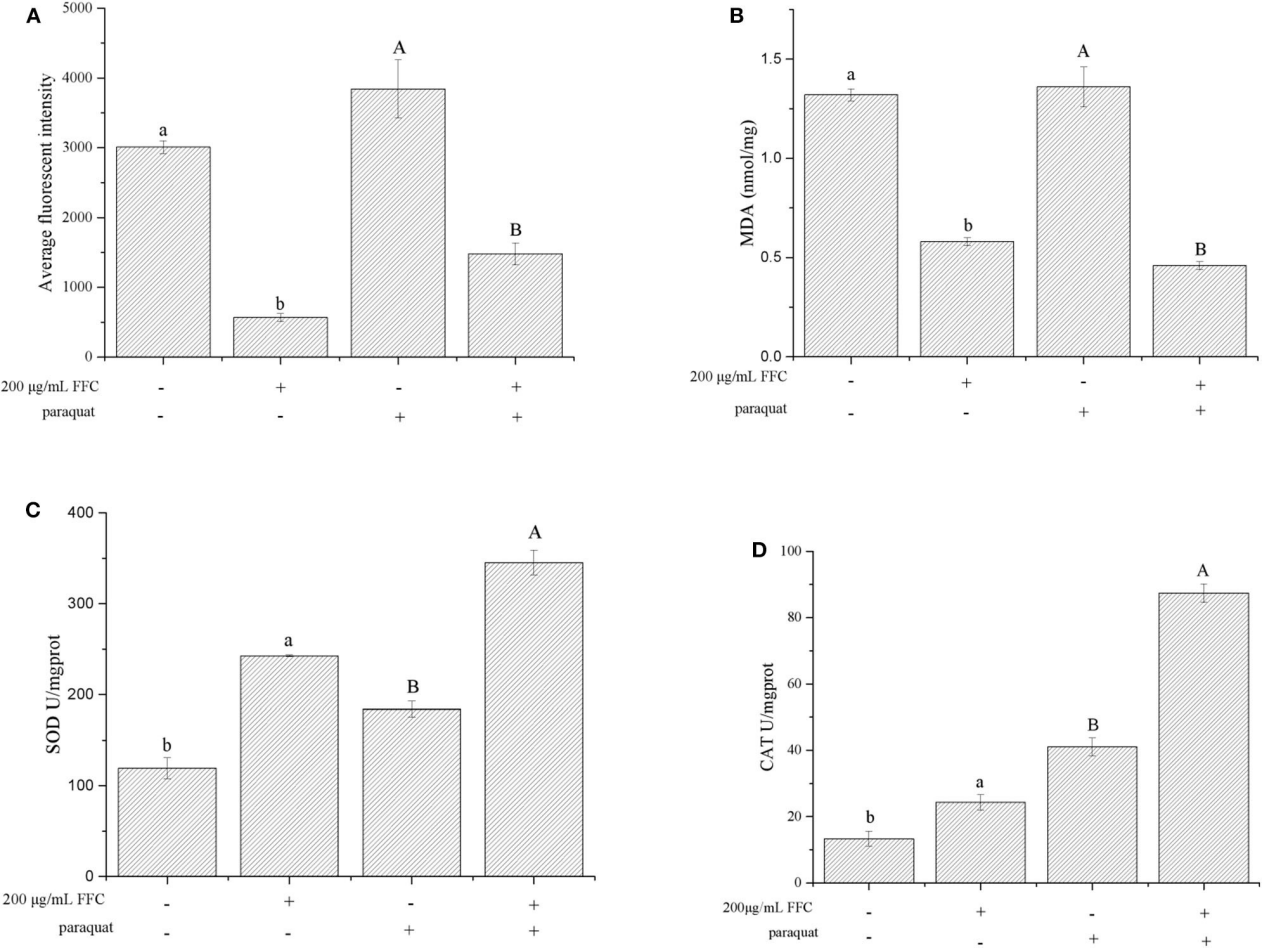
Effects of the purified FFC on the accumulation of ROS and MDA and enzyme activity under normal and 25 mg/mL paraquat-induced oxidative stress conditions. **(A)** ROS, **(B)** MDA, **(C)** SOD, and **(D)** CAT were measured according to the methods described in Measurement of ROS and Measurement of SOD, CAT, and MDA, in which 80 or 200 worms were collected for sample preparation after treatment with the purified FFC or control for 96 h. SOD and CAT enzyme activity was expressed as units/mg of protein. Data are expressed as the mean ± SD of three independent experiments (*N* = 3). Different letters in these groups denote values that were significantly different (*p* < 0.05).

Antioxidants have the potential to reduce oxidative stress levels, thereby delaying aging and age-related diseases (50). The accumulation of ROS accelerates aging and mitochondrial damage (51). In addition, the amount of MDA can reflect the degree of lipid peroxidation in the body, indirectly reflecting the degree of cell damage. Therefore, we investigated whether FFC could affect the accumulation of ROS and MDA under normal and oxidative stress. The accumulation of ROS was measured using H_2_DCF-DA, and the fluorescent dye DCF (2′,7′-dichlorofluorescein) indicated the accumulation of ROS in the cells. Under normal and 25 mg/mL paraquat-induced oxidative stress conditions, the ROS accumulation of the worms fed with the purified FFC were 81.07% and 61.46% lower than those of the control, respectively (Figure 6A). Similarly, the purified FFC could significantly decrease the amount of MDA in the worms under normal and paraquat-induced oxidative stress conditions (*p* < 0.05), which was 56.06 and 66.18% lower than the untreated worms, respectively (Figure 6B). These findings indicated that lifespan extension of the worms by FFC might be partly related to its ability to scavenge intracellular ROS and MDA.

SOD plays an extremely important role in preventing aging and biomolecular damage, and the activity of the enzyme in organisms reflects the magnitude of the antioxidant capacity (52, 53). On the other hand, CAT can react with H_2_O_2_ in organisms and decompose it to H_2_O and O_2_, thereby preventing the damage to the body from oxygen metabolisms and protecting living tissues from poisoning (54). To investigate the effect of FFC on worm's antioxidant enzymes, the activity of SOD and CAT in FFC treated and untreated worms were examined under normal and oxidative stress conditions (exposure to 25 mg/mL paraquat). The activity of SOD enzyme in the worms under the presence of the purified FFC was significantly higher than that of the untreated worms (*p* < 0.05) as depicted in Figure 6C, which was increased by 103.14 and 87.56% respectively under normal and oxidative stress conditions compared to the control. Similarly, the CAT enzyme activity of the worms fed with the purified FFC was also evidently higher than that of the control, and was 81.91 and 113.07% higher than the control worms accordingly (Figure 6D). These results suggested that FFC could increase SOD and CAT enzyme activity of *C. elegans* under both normal and oxidative stress conditions, indicating that lifespan elongation of *C. elegans* exposed to FFC might attribute to the elimination effect of FFC on the excess ROS and MDA by improving the enzyme activity of SOD and CAT. Similar results were reported in the study of Zhou et al. (55), who discovered that flavonoids from the herb *Scutellariae barbatae* could delay the aging of *C. elegans* through up-regulating the activity of antioxidant enzymes like SOD and CAT.

## Conclusion

Taken together, we analyzed five major peak components in the purified FFC and identified three of them by HPLC and UPLC-MS/MS as diosmetin-6-8-di-*C*-glucoside (peak 1), hesperidin (peak 3), and diosmetin-6-*C*- glucoside (peak 4), while Peaks 2 and 5 were preliminarily judged as limocitrol 3-alpha-l-arabinopyranosyl-(1->3)-galactoside and scutellarein 4′-methyl ether 7-glucoside by molecular structure correlation, respectively. Further, the antioxidant and antiaging activities of the purified FFC were evaluated *in vivo* and *in vitro*. Our findings suggested that the purified FFC had excellent prospects for its antioxidant function *in vitro*. In addition, the purified FFC could increase the lifespan of *C. elegans* without causing side effects on their physiological functions including reproduction, locomotion ability, sinusoidal locomotion, and head swing frequency. Also, the purified FFC could prolong the lifespan of *C. elegans* by enhancing its resistance to thermal and oxidative stress. A further study showed that the lifespan improvement mediated by the purified FFC under normal and oxidative stress conditions was associated with the decreased ROS and MDA accumulation and the increased SOD and CAT enzyme activities. These results provided useful information for the utilization of FFC in natural antioxidant and antiaging functional foods.

## Data Availability Statement

The raw data supporting the conclusions of this article will be made available by the authors, without undue reservation.

## Author Contributions

XL: data curation and writing - original draft. JW: methodology and investigation. HC, MS, and QZ: writing - review and editing. AZ: conceptualization, writing - review and editing, and supervision. HC: supervision. YC: investigation and supervision. All authors contributed to the article and approved the submitted version.

## Conflict of Interest

HC was employed by the company Guangdong Zhancui Food Co., Ltd. The remaining authors declare that the research was conducted in the absence of any commercial or financial relationships that could be construed as a potential conflict of interest.

## Abbreviations

FFC: flavonoids of finger citron
*C. elegans*: *Caenorhabditis elegans*
*E. coli* OP50: *Escherichia coli* strain OP50
DPPH: 2,2-diphenyl-1-picrylhydrazyl
ABTS: 2,2-azinobis (3-ethyl-benzothiazoline-6-sulphonic acid) diammonium salt
AAPH: 2,2′-azobis (2-methylpropionamidine) dihydrochloride
H_2_DCF-DA: 2′ ,7′-dichlorodihydrofluorescein diacetate
DCF: 2′ ,7′-dichlorofluorescein
ROS: reactive oxygen species
MDA: malondialdehyde
SOD: superoxide dismutase
CAT: catalase.
